# Environmental Stressors on Skin Aging. Mechanistic Insights

**DOI:** 10.3389/fphar.2019.00759

**Published:** 2019-07-09

**Authors:** Concepcion Parrado, Sivia Mercado-Saenz, Azahara Perez-Davo, Yolanda Gilaberte, Salvador Gonzalez, Angeles Juarranz

**Affiliations:** ^1^Department of Histology and Pathology, Faculty of Medicine, University of Málaga, Málaga, Spain; ^2^Cantabria Labs, Madrid, Spain; ^3^Dermatology Service, Miguel Servet Hospital, Zaragoza, Spain; ^4^Medicine and Medical Specialties Department, Alcala University, Madrid, Spain; ^5^Biology Department, Sciences School, Autonoma University, Madrid, Spain

**Keywords:** skin aging, air pollutant, oxidative stress, inflammation, photo-pollution

## Abstract

The skin is the main barrier that protects us against environmental stressors (physical, chemical, and biological). These stressors, combined with internal factors, are responsible for cutaneous aging. Furthermore, they negatively affect the skin and increase the risk of cutaneous diseases, particularly skin cancer. This review addresses the impact of environmental stressors on skin aging, especially those related to general and specific external factors (lifestyle, occupation, pollutants, and light exposure). More specifically, we have evaluated ambient air pollution, household air pollutants from non-combustion sources, and exposure to light (ultraviolet radiation and blue and red light). We approach the molecular pathways involved in skin aging and pathology as a result of exposure to these external environmental stressors. Finally, we reflect on how components of environmental stress can interact with ultraviolet radiation to cause cell damage and the critical importance of knowing the mechanisms to develop new therapies to maintain the skin without damage in old age and to repair its diseases.

## Introduction

The skin is the first defensive barrier of our body being in contact with the environment. All the cells and layers of the skin are involved in the barrier defense. However, some environmental factors could make the skin vulnerable. It is well-documented that sunlight can produce oxidative stress, photoaging, and photocarcinogenesis (Birch-Machin and Bowman, 2016) and more recently, research is focused on the assessment of injury that environmental contaminants may cause to the skin (Krutmann et al., 2017; Salthammer et al., 2018).

Environmental factors that influence skin aging and carcinogenesis fall into the following major categories: sun radiation (ultraviolet radiation, visible light, and infra-red radiation), air pollution, tobacco smoke, nutrition, some less well-studied factors, and cosmetic products.

Air pollutions are of more significant interest for also having a possible synergistic effect with solar radiation. As new data reported by the World Health Organization (WHO) reported in 2018, nine out of 10 people breathe high levels of air pollutants (World Health Organization, 2018). The deaths per year due to ambient (outdoor) air pollution reached 4.2 million in 2016. Outdoor air pollution originates from natural and anthropogenic sources. Natural sources produce pollution in areas where hazards could exist, creating forest fires, natural disasters, and dust torments. However, pollution caused by anthropogenic sources is overcome by contamination from natural sources. The WHO considers six types of air pollutants: particulate matter, nitrogen dioxide, sulfur dioxide, black carbon, carbon monoxide, and ground-level ozone (World Health Organization, 2012).

In this review, we analyze the effects of environmental stressors in skin aging and specifically the air pollution oxidative stress effects in premature aging.

## Skin Aging and Oxidative Stress

Skin protection is affected by aging that produces inflammation, disruption of the skin barrier, decreased ability to repair wounds, and increased risk of skin cancer. In the skin aging also a reduction of elasticity, pigmentation, and harmful effects in the extracellular matrix (ECM) have been found (Krutmann et al., 2017).

Several types of cellular stressors are implicated in skin aging, including: i) telomere shortening; ii) changes in chromatin structure; iii) activation of oncogenes; iv) mitochondrial dysfunction; v) oxidative stress; and vi) epigenetic strands (Wang and Dreesen, 2018).

The appearance and function of human skin undergo profound changes with increasing chronological age (intrinsic or chronological aging) and with cumulative exposure to external factors (extrinsic aging).

Skin aging is characterized by the appearance of fine wrinkles, the loss of elasticity, deleted repair wounds, age spots, and the loss of skin tone. Chronological aging leads to a thinning of the epidermal and dermal layers as well as loss of sensitivity due to the decrease in the production of sex hormones and the number of nerve endings. Photoaged skin has deeper wrinkles, a more significant loss of elasticity compared with intrinsic aging, and a rough texture (Montagna et al., 1989). Exposure to ultraviolet radiation (UVR) is related to the appearance of skin cancer.

Most of the events that contribute to intrinsic and extrinsic aging, such as mitochondrial dysfunction, inflammation, and the abnormality of extracellular matrix homeostasis, are a consequence of oxidative stress (Kammeyer and Luiten, 2015; Lephart, 2016). In intrinsic aging, reactive oxygen species (ROS) are produced through cellular oxidative metabolism. Exposure to solar radiation also increases the production of ROS and causes damage to DNA, proteins, and lipids and reduces antioxidant levels in the skin (Kammeyer and Luiten, 2015).

### Chronological Skin Aging

Skin that is aged only by intrinsic factors virtually doesn’t exist. In general, individuals wear a skin that reflects several stages of extrinsic aging, superimposed on the level of intrinsic aging.

The intrinsic or chronological aging is a multifactorial phenomenon in which several factors such as genetics and metabolism are involved (Poljšak and Dahmane, 2012; Quan et al., 2015). In the skin, around 1.5–5% of the oxygen consumed is converted into ROS by intrinsic processes (Poljšak and Dahmane, 2012), being ROS essential factors in intrinsic aging.

Keratinocytes and fibroblasts are the primary sources of mitochondrial ROS in the skin. Melanogenesis also produces hydrogen peroxide and ROS causing melanocytes are under oxidative stress of high degree. (Slominski et al., 2004; Slominski et al., 2012a). The production of ROS in the mitochondria of keratinocytes occurs in the deepest cells of the epidermis, especially in the stem cells at the basal layer; in the final process of differentiation, the keratinocytes stratum corneum lose their organelles. However, ROS are also produced in the endoplasmic reticulum (ER) being members of the cytochrome P450 family, the main ROS producers. The family of cytochrome P450 is responsible for the detoxification of xenobiotics or lipophilic compounds (Rinnerthaler et al., 2015). Finally, also the cytosol produces ROS as a product of the metabolism of arachidonic acid. The lipoxygenase and cyclooxygenase enzymes use arachidonic acid to synthesize prostaglandin (PG) and the leukotrienes, respectively. Both proteins produce superoxide in the presence of the nicotinamide adenine dinucleotide (NADH) and nicotinamide adenine dinucleotide phosphate (NADPH). Arachidonic acid levels are relatively low in the skin but they increase in skin aging (Rinnerthaler et al., 2015).

As the skin ages, and due to the imbalance in the production of free radicals, the number of keratinocytes and fibroblasts decreased as a consequent reduction in the turnover of the epidermis and the subsequent decrease in collagen and proteoglycans, respectively. All this leads to atrophy and contributes to fibrosis. These changes may also lead to increased production of free radicals (Tulah and Birch-Machin, 2013).

Another crucial factor in skin aging is the increased expression of matrix metalloproteinases (MMPs) and decreased expression of MMP inhibitors (TIMP). These alterations may be related to the production of ROS. The increase of ROS up-regulated the activator protein-1 (AP-1), which suppresses the transforming growth factor β (TGF-β) receptors, which in turn blocks the synthesis of collagen (Quan et al., 2015). Activation of AP-1 stimulates collagen degrading by MMP and activates the nuclear factor kappa B (NF-κB), an activator of the inflammatory response (Poljšak and Dahmane, 2012). The increasing inflammatory response with overexpression of cytokines and interleukins stimulates the production of ROS (Quan et al., 2015).

### Photoaging

**Figure 1:**
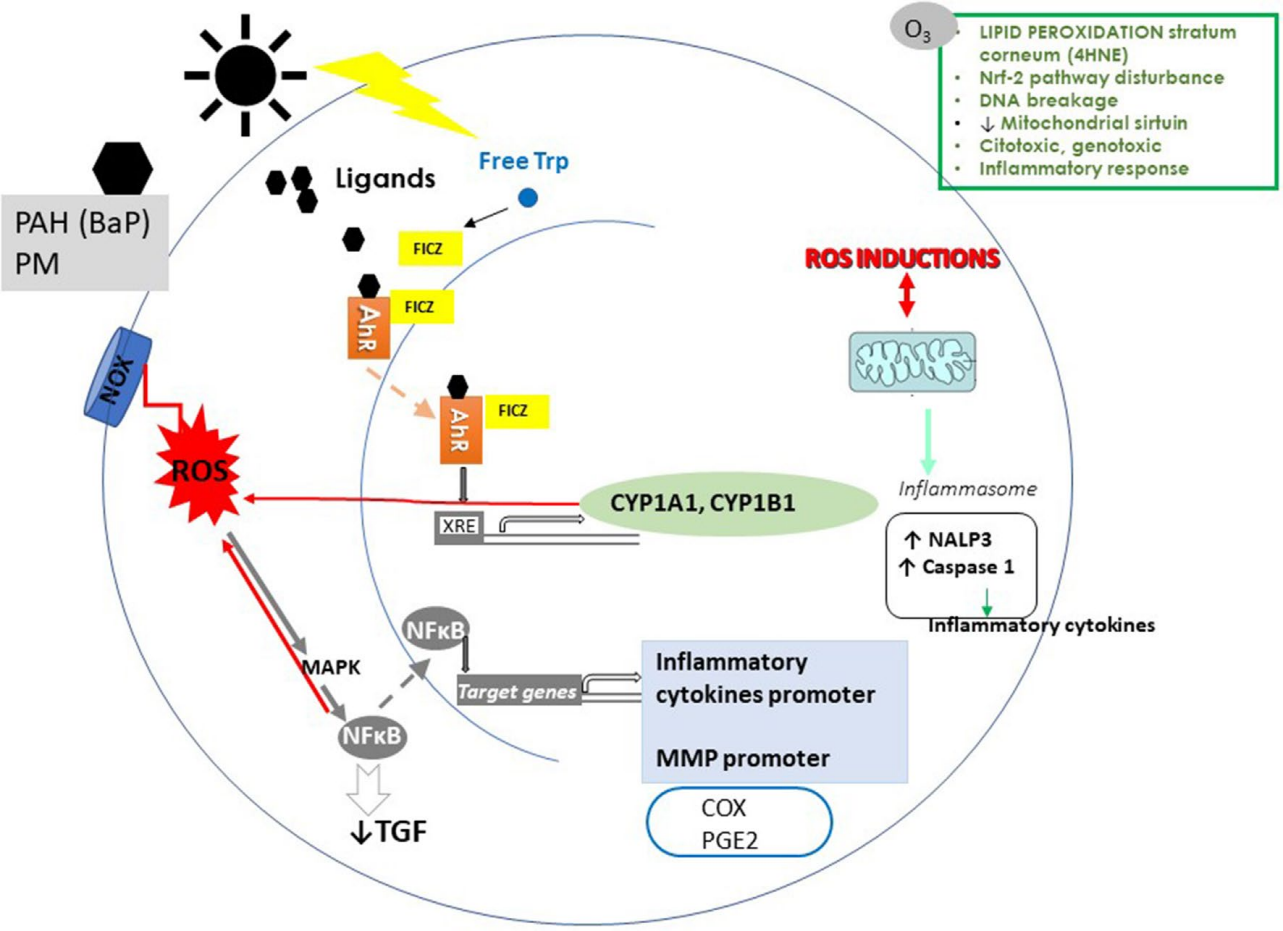
Effects of air pollution and ultraviolet radiation (UVR) on the skin. A simplified representation of the effects of epidermal keratinocyte effects of particulate matter (PM), polycyclic aromatic hydrocarbons (PAH), ozone (O_3_), and UVR. The AhR binds to PM, PHA, O3, and UVR adducts. After ligand binding, AhR translocates to the nucleus and induces the transcription of cytochrome P450 (CYP 1A1, CYP 1B1). CYP 1A1 generates toxic and reactive intermediates of xenobiotics, increasing ROS. In addition to the canonical AhR pathway, the activation of AhR affects NF-κB pathways. NF-κB induces an increase of proinflammatory cytokines and MMP and decreases TGF-β, and I collagen synthesis. ROS also induces NF-κB activation. Among the next targets of the pollutants are the proinflammatory mediators (COX and PGE2). Pollutants induce the production of NALP3a. NALP3 stimulates caspase-1 to promote proinflammatory cytokines. In epidermal KC, AhR activation results from the absorbance of UVB radiation by tryptophan (Trp). Trp induces the generation of 6-formylindolo[3,2-b]carbazole (FICZ). FICZ is a high-affinity ligand for AhR. ROS induced by pollutants alters lipids, proteins, and DNA. Also, oxidative stress causes overexpression of MMP in the ECM and collagen degradation.

Oxidative stress is the primary cause of extrinsic aging or photoaging caused mainly UVR. Other environmental extrinsic factors also cause skin aging (Krutmann et al., 2017). The principal effects of UVR in the skin are DNA damage, oxidative stress, deleterious impact on the ECM, inflammation, and immunosuppression (Kochevar et al., 2012; Zamarrón et al., 2018) (**Figure 1**). The UVR effect on the skin also includes activation of the neuroendocrine system by nerve transmission or by cutaneous originating chemical mediators (see the review Slominski et al., 2012b; Slominski et al., 2018a). Environmental stress, in the skin, as occurs in photoaging, can produce activation of the hypothalamic–pituitary–adrenal (HPA) axis either by nervous route or by humoral factors. This activation induces homeostatic responses to counteract the damage and systemic (Slominski and Wortsman, 2000; Slominski et al., 2013a; Slominski et al., 2013b; Slominski et al., 2015). Oxidative stress is the leading cause of photoaging, and it is involved in several diseases, including cancer (Kammeyer and Luiten, 2015). UVR, mostly UV-B radiation, alters DNA by the formation of pyrimidine–pyrimidine dimers (mainly thymine–thymine dimers). Those dimer creations are the basis of mutations in specific genes, including the tumor suppressor gene p53. UV-B radiation also damages DNA through ROS promoting the generation of 8-hydroxy-2′-deoxyguanosine (8-OH-dG), a marker of DNA oxidative damage (Kochevar et al., 2012). However, 8-OH-dG is the major UVA-induced DNA lesion (Kammeyer and Luiten, 2015).

The inflammatory effect of UVR is due to the oxidation of the membrane lipids and the production of arachidonic acid. Cyclooxygenase enzymes (COX) convert arachidonic acid into PG, amplifying the recruitment of inflammatory cells. Besides, other pro-inflammatory cytokines are activated ROS as interleukin-6 (IL-6). ROS also induce the activation of tumor necrosis factor-α (TNF-α) and NF-κB expression. In addition, UVR promotes peroxidation by ROS, which causes the expression of inducible nitric oxide synthase (iNOS) that mediates angiogenesis and hyperpermeability by up-regulation of the vascular endothelial growth factor (VEGF) (Christensen et al., 2017).

UVR also induces immunosuppression and immunological tolerance. A marked decrease in the numbers of epidermal Langerhans cells and isomerization of the urocanic acid [3-(1H-imidazole-4-yl)-2-propenoic acid; UCA] have been described to be induced by UVR. The isomerization of trans-UCA to the cis-isomer represents a signal of immune suppression. Also, the immunosuppressive effects of UVR are due to the actions of immunomodulatory molecules such as TNF-α, prostaglandin PG E2, and IL-10 (Christensen et al., 2017).

The excess of ROS induced by UVR also activates the mitogen-activated protein kinases (MAPKs) and NF-κB, and finally increases the transcription of the MMPs. The increase of MMPs causes dysregulation of the ECM homeostasis and the degradation of collagen and elastin (Kochevar et al., 2012). But not only ultraviolet radiation produces oxidative stress and aging. Also, infrared radiation (IR), used as therapeutic effects at low doses, has a harmful impact on the skin related to photoaging. While IR-B and IR-C only induce effects in the upper layers of the skin, IR-A penetrates deeper into the skin (Albrecht et al., 2019). IR-A generates mitochondrial ROS, followed by an increase in MMP-1 through the activation of the MAPK. It was also shown that the IR-A leads to overexpression of MMP-9 without an increase in TIMP expression. Finally, IR-A decreases the synthesis of collagen, promotes angiogenesis, and increases the number of mast cells, events associated with skin aging (also, as observed in aging, IR-A decreases the turnover of keratinocytes and the cellular density of epidermal Langerhans cells, which influences the repair of the wound). It also alters the levels of TGB-β (Slominski et al., 2018a).

In the same way and until a few decades ago, the visible light (VIS) did not seem to induce photobiological effects on the skin. The works of Pathak in 1962 (Pathak et al., 1962) and those of Kollias and Baqer in 1984 (Kollias and Baqer, 1984) made it possible to hypothesize that VIS could contribute to photo-aging. Both authors appreciated hyperpigmentation after subjecting the skin to VIS. Exposure of human skin to different doses of VIS, from 40 to 180 J/cm^2^, produced ROS in a dose-dependent manner. VIS also produces DNA damage indirectly through ROS production (Slominski et al., 2018a).

In human skin, VIS plus IR increases MMP-1 and MMP-9 expression, decreases type I procollagen levels, and recruits macrophages to the irradiated site. Visible blue-violet light degrades carotenoids and generates free radicals and ROS. Both UVA and VIS induced pigmentation in skin types IV–VI, but the pigmentation induced by VIS was darker and more sustained. These findings have potential implications and clinical relevance because of the possible role of VIS in the pathogenesis of photo-induced pigmentary disorders (Zamarrón et al., 2018).

### Skin Barrier and Aging

The cutaneous barrier (microbiome, immune barriers, chemicals, and physical factors) allows protection to the body and maintains skin homeostasis. The epidermis exerts the barrier functions of the skin, and the stratum corneum (SC) is mostly responsible for these barrier functions. Dry skin occurs on aged skin with redness and itching. These signs on aged skin are related to impaired enzymatic processes, partly due to the decrease in the water content of SC (Tončić et al., 2017).

The tight junctions between the keratinocytes, anchored in their cytoskeleton, contribute to the physical barrier. The chemical barrier consists of lipids, antimicrobial peptides, and degradation products of filaggrin (FLG). The intercellular lipid determines the permeability of the stratum corneum. In addition, the surface of the skin is covered by a lipid film produced by the sebaceous glands in the dermis that is secreted through the pilosebaceous follicles. Human sebum is mainly composed of squalene, wax monoesters, and triglycerides. This permeability barrier also protects against potentially toxic substances from the environment. Some of the stratum corneum lipids also serve as substrates for the enzymatic production of potent antimicrobials (Wertz, 2018). The immunological barrier and antimicrobial defense are aspects of great importance in the cutaneous barrier. Also, the skin is colonized by an abundant and diverse community of microbes, known as “microbiome” (Dąbrowska et al., 2018). The cutaneous microbiome plays an essential role in the cell cycle of the keratinocytes and in the immune networks of the skin that have systemic implications.

The aged epidermis increases susceptibility to irritant contact dermatitis and, often, severe xerosis, and impaired permeability to the drug, suggesting alterations of the barrier (Dąbrowska et al., 2018). Thus, in the aging of the skin, its barrier function is diminished, with high protease activity and a decreased production of natural moisturizing factor (NMF) formed from the proteolysis of FLG.

With aging, cellular replacement in the skin and mechanical protection continually decreases. The healing of wounds and immune responses is delayed. Likewise, thermoregulation, the production of sweat, and the creation of sebum are compromised. Also, the proliferation and desquamation of keratinocytes are reduced (Rinnerthaler et al., 2015).

### The Aryl Hydrocarbon Receptor in Skin Aging

The aryl hydrocarbon receptor (AhR) is a cytosolic ligand-activated transcription factor expressed in the skin (keratinocytes, fibroblasts, and melanocytes) as well as in regulatory T cells. AhR contributes to physiological and physiopathological processes in the skin and regulates cellular proliferation, inflammation, and melanogenesis (Eckers et al., 2016).

AhR can be activated for a large variety of endogenous or exogenous ligands. The AhR exogenous ligands are abundant and include environmental ozone (O_3_) as well as other air pollutant chemicals such as particulate matter (PM) and persistent organic pollutants (POPs) such as dioxin. In its inactive state in the cytosol, AhR forms a protein complex. After ligand binding, AhR is translocated to the nucleus and mediates numerous biological effects by inducing the transcription of several genes. The AhR target genes encode drug-metabolizing enzymes such as cytochrome P450 (CYP) 1A1, 1A2, and 1B1 as well as proteins that control cell division, apoptosis, and cell differentiation (Esser et al., 2018) (**Figure 1**).

CYP enzymes are phase 1 metabolizing enzymes that catalyze biotransformation of xenobiotics as polycyclic aromatic hydrocarbons (PAH); however, this biochemical process sometimes generates toxic and reactive intermediates of xenobiotics, which misbalance redox homeostasis, increasing ROS (Larigot et al., 2018). On the other hand, AhR in the non-canonical pathway also plays a critical role in regulated T cell responses. Some AhR ligands modulate the regulatory T cells differentiation or increase the proinflammatory differentiation of Th 17. Therefore, activation of AhR is critical in the increase of proinflammatory responses (O’Driscoll and Mezrich, 2018). In addition to the canonical AhR pathway, the activation of AhR often affects other signal transduction pathways, including NF-κB signals (Pollet et al., 2018).

AhR may serve as a sensor for UVR, and its formation explains cutaneous as well as extracutaneous induction of CYP1A1 enzyme activity after skin exposure to UVR (Eckers et al., 2016).

Conversely, the activation of AhR in chronic inflammatory skin diseases, such as atopic dermatitis (AD) and psoriasis, is entirely different from activation in healthy skin. In AD, activation of AhR exerts anti-inflammatory activity by preventing inflammation of the skin. Therefore, the alteration of the AhR signaling in the skin can have detrimental or beneficial effects, depending on the disease, the participating cells, and the activity of the immune system (Haarmann-Stemmann et al., 2015). Thus, in 2018, the fourth AhR meeting concluded that the normal physiological AhR continues to present different results due to its highly complex and tissue-specific functions. Some examples of its tasks are autoimmunity, metabolic imbalance, inflammatory skin diseases, gastrointestinal diseases, cancer development, and perhaps aging. Accurate knowledge of AhR signaling can generate novel therapies (Esser et al., 2018).

## Skin Aging and Air Pollution

The increase in air pollution has harmful effects on human skin. The skin is exposed to environmental air pollutants, such PAHs, oxides, volatile organic compounds (VOCs), PM, and O_3_. Although human skin acts as a biological barrier against pro-oxidative air pollutants, prolonged exposure to their high levels can induce alterations in skin homeostasis and has been associated with aging as well as inflammatory skin conditions (AD and eczema). The risk of skin cancer induced by air pollution is one of the central health fundamental problems from environmental studies (Mancebo and Wang, 2015; Krutmann et al., 2017).

**Figure 2:**
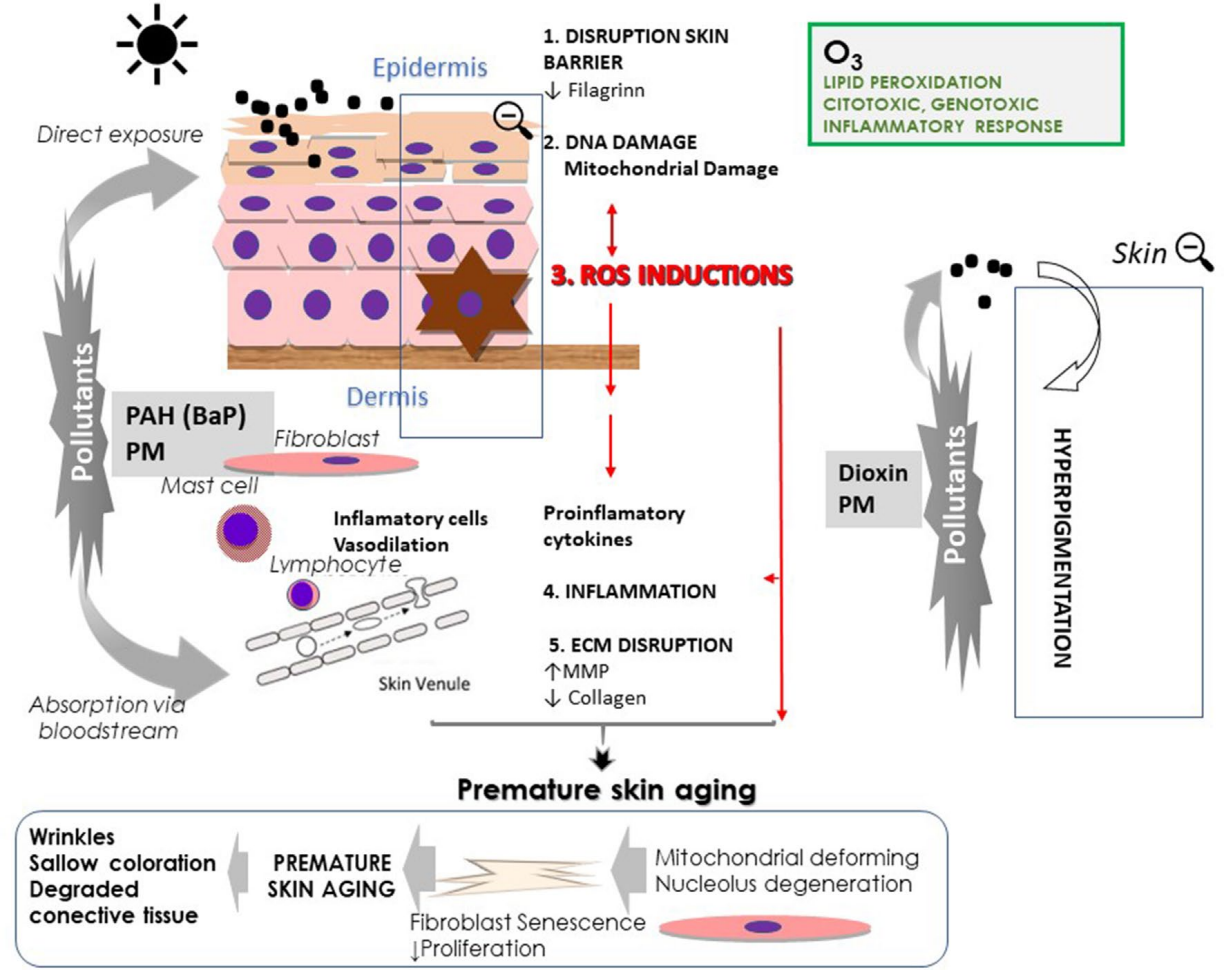
Skin responses to air pollutions (particulate matter, PM; polycyclic aromatic hydrocarbons PAH; and ozone, O_3_) and ultraviolet radiation.

Three mechanisms seem to be involved in the adverse effects of ambient air pollutants on skin health: a) the generation of free radicals; b) the induction of an inflammatory cascade; and c) the impairment of the skin barrier (**Figure 2**). Thus, chronic exposure to O_3_ produces oxidative damage in the stratum corneum, which generates free-radical species. Also, O_3_ depletes the reserves of both enzymatic and non-enzymatic antioxidants in the skin and decreases vitamin C and E levels. In the mitochondria, O_3_ decreases ATP and sirtuin 3, a protein related to the elimination of free radicals. Also, the other air pollutants promote oxidative stress and a proinflammatory environment in the skin. The inflammatory mediators activate the chemotaxis of granulocytes and phagocytosis. The response to environmental stressors also activates cutaneous and central neuroendocrine responses. Epidermal keratinocytes, Langerhans cells, melanocytes, and dermal cells such as fibroblasts, macrophages, mast cells, and lymphocytes may be involved in skin cutaneous neuroendocrine responses. On the other hand, extrinsic factors can alter skin immunity and the immune system of skin interacts with the neuroendocrine responses (Slominski et al., 2012b; Slominski et al., 2018a). Ultimately, all the processes previously related produce direct and indirect toxicity to the skin (Mancebo and Wang, 2015; Krutmann et al., 2017).

As mentioned earlier, WHO (World Health Organization, 2012) considers six types of air pollutants, but the six types could be classified attending to the source in four groups: particulate matter (PM), persistent organic pollutants (POP) (e.g., dioxins), gaseous pollutants (e.g., CO, SO2, NOx, VOCs, O_3_), and heavy metals (e.g., lead, mercury).

### Particulate Matter

Particulate matter is a common indirect indicator of air pollution and constitutes one of the most critical problems for air quality and climate changes. The literature on atmospheric PM has increased enormously over the last 10 years due in part to the advances in technologies that have allowed for a better understanding of their chemical and physical properties (van Donkelaar. et al., 2019).

PM affects more people than any other air pollutants. Chronic exposure to PM increased the risk of developing respiratory and cardiovascular diseases, as well as cancer (Lelieveld et al., 2015; Hamanaka and Mutlu, 2018). In this regard, particles with a diameter of 10 µm or less (PM_10_) can penetrate deeply in the lung, being the particles most dangerous to health those with a diameter of 2.5 µm or less (PM_2.5_). PM_2.5_ can pass the pulmonary barrier and reach the blood. In 2012, the WHO estimated that an average annual reduction in PM_2.5_ equal to or less than 35 μm/m^3^, something that is very common in many cities, could reduce deaths related to air pollution by around 15% (World Health Organization, 2012).

PM consists of a mixture of components that may be liquid and solid as well as organic and inorganic. Although PM changes its physical and chemical characteristics depending on the location, common chemical constituents of PM include sulfates, nitrates, ammonium, organic carbon, particle-bound water, metals, and PAH (Hopke and Rossner, 2006; World Health Organization, 2014). The WHO references the levels of PM in μg/m^3^ annual and 24-h averages. The reference values for PM_10_ are 20 and 50 μg/m^3^, respectively, and 10 and 25 μg/m^3^, respectively, for PM_2.5_ (World Health Organization, 2014).

Recently, ultrafine particles (UFPs) (≤100 nm in diameter) that are abundant in urban air have been considered to be a health risk having a more significant potential for adverse effects for humans since they can penetrate the bloodstream and accumulate in the lungs and other organs of the body (Krutmann et al., 2014). The examples of UFP are diesel exhaust particles, cooking products, heating, and wood burning in indoor environments. However, there are no reference values for them.

Not only does PM affect the lung or cardiovascular system, but there is also a relationship with exposure to PM skin diseases. Today, there is more evidence available on the effects of PM on skin damage including skin aging (Vierkötter et al., 2010; Krutmann et al., 2017; Ngoc et al., 2017). The small PM can pass into the circulation and damage the dermis, without alterations to the epidermis. On the other hand, PM also causes alterations to the skin barrier. The transdermal passage is also accepted, starting from the hair follicles and sweat glands or through the stratum corneum (Magnani et al., 2016).

The specific components of the PM and their concentrations are not well understood (O’Driscoll et al., 2019). Several researchers have been made to identify the effects of toxic effect of each component, PAH. PAHs are ubiquitous environmental toxicants and AhR ligands. However, until now, only existing air quality standards focus exclusively on benzo(a)pyrene (B[a]P), a member of PAH, bound to the PM_10_ fraction (Pehnec and Jakovljević, 2018), with few results of the effects of PAH binding to PM in human health.

In our review, we will deal with PM_10_, PM_2.5_, and UFP effects on the skin. PAH has the characteristics of POP and PAH will be discussed in the next section, with particular references to 1 of the 16 PAHs with carcinogenic power, such as B[a]P (Idowu et al., 2019).

#### Particulate Matter and Oxidative Stress/Inflammation

PM causes damage to the skin surface and even in the dermis in the case of PM with a smaller diameter or some of the PM compounds that can reach the dermis *via* blood circulation.

Several studies *in vivo* and *in vitro* support the idea that PM induces skin damage by producing oxidative stress and inflammation, and both mechanisms are essential contributors to skin aging (**Table 1**, **Figure 1**). Exposure of the skin to PM induces lipid peroxidation, ROS, DNA damage, apoptosis, and extracellular matrix damage (Magnani et al., 2016; Lee et al., 2016a; Zhang et al., 2017a; Idowu et al., 2019). PM binds to AhR and induces the intranuclear signaling pathway, favoring ROS formation and proinflammatory gene expression (Esser et al., 2018) (see the section The Aryl Hydrocarbon Receptor (AhR) in Skin Aging).

**Table 1:** Effects of Particulate matter (PM) in the skin.

Skin effects	Pollutants	Type of study	Skin damage	Mechanism skin damage	References
**Oxidative stress/inflammation**	PM_10_	*In vitro*	↑ROS	↑NOX ↑COX-2/PGE2	(Lee et al., 2016a)
		*In vitro*	Inhibited proliferation	↑AhR-MAPK signalling ↑CYP1A1 ↑MMP1 ↑Genes of cell aging.	(Qiao et al., 2017)
	PM_2.5_	*In vitro*	↑ROS ↑IL-1β ↑IL-6	↑Cryopyrin/NALP3 ↑NF-κB	(Zhang et al., 2017b)
		*In vitro*	↑ROS	↑TLR5 ↑NOX4	(Ryu et al., 2019)
	PM 5.85µm	*In vitro*	↑ROS ↑IL-8, ↑IL-1β	↑TPRV ↑NF-κB	(Kwon et al., 2018)
	PM_10_	*In vivo* BALB/c nude mice	Inflammation in skin	↑AhR ↑NOX ↑COX-2/PG-2	(Lee et al., 2016a)
**Skin barrier dysfunction**	PM_10_	*In vitro*	↓Filaggrin	↑AhR ↑COX2/PGE2.	(Lee et al., 2016b)
	PM_2.5_	*In vitro*	↓KRT 10 ↓Desmocollin ↓Claudin 1	↑CYP1A1 CYP1B1 ↑COX2 ↑NF-κB	(Pan et al., 2015)
		*In vitro*	↓KRT 16	↑Caspase 3 ↑Bax	(Nguyen et al., 2019)
			↓Keratinocyte proliferation ↑Keratinocyte apoptosis	↑p53	
		*In vivo* BALB/c mice	Dermal inflammatory cells infiltration		(Jin et al., 2018)
**Extracellular matrix dysfunction**	PM_10_	*In vitro*	↑MMP-1 ↑MMP-3 ↓ Collagen	↑CYP1A1, CYP1B1 ↓TGF-β	(Park et al., 2018)

AhR, aryl hydrocarbon receptor; CYP1A1, CYP1B1, cytochrome P450 (CYP1A1, CYP1B1); COX, cyclooxygenase; IL, interleukin; KRT, keratin; MAPK, mitogen-activated protein kinases; MMP, matrix metalloproteinase; NF-kB, nuclear factor kappa B; Nrf2, nuclear factor erythroid 2-like 2; NOX, NADH-oxidase; PGE, prostaglandin E; ROS, reactive oxygen species; TGF-β, transforming growth factor β; TLR, toll-like receptors; TNF-α, tumor necrosis factor α.

PM_2.5_ also induces, *in vitro*, the production of the sensor of stress, cryopyrin (also called NALP3a), that is activated by ROS (Zhang et al., 2017b). NALP3a is a member of the nucleotide-binding oligomerization domain-like receptor (NLR) and senses stress or danger and induces ROS (Martinon, 2010). Furthermore, NALP3 stimulates caspase-1 activation to promote the processing and secretion of proinflammatory cytokines as IL-1β and IL-6 (Zhang et al., 2017b) (**Figure 1**). By TEM, PM_2.5_ has been found inside HaCat cells in the mitochondria, cytoplasm, and nucleus also providing a molecular insight into PM_2.5_-induced skin injury (Zhang et al., 2017b) (**Table 1**). However, a model of senescence in keratinocytes and fibroblast induced by PM has been described (Qiao et al., 2017). The PM (SRM1649b, a model of exposure to ambient urban PM urban dust, average diameter 10.5 μm) increases aging-related genes after the activation of AhR-MAPK signaling. AhR-specific antagonists reverted the aging in skin cells and could be useful in preventing skin aging and associated diseases such as non-melanoma skin cancer (Qiao et al., 2017) (**Table 1**) (**Figure 1**).

Recently, the action of PM 2.5 containing a high content of heavy metals has been established. In raw 264.7 macrophage cells exposed to this type of PM 2.5, an increase in ROS, productions, and TNF-α was observed. Exposure to nanoparticles containing melatonin suppressed the deleterious effect of PM 2.5 by suppressing the ROS and TNF-α level (Zhang et al., 2017a). Melatonin under conditions of oxidative stress acts as a local antioxidant, decreasing mitochondrial damage. Melatonin also prevents free radicals from damaging proteins so it can be considered as an agent that prevents intrinsic skin aging and photo aging. Melatonin can also protect DNA against free radicals, not only by eliminating hydroxyl radicals or by increasing gene expression of antioxidant enzymes, but also by regulating genes involved in the repair of damage to DNA (Slominski et al., 2014; Skobowiat et al., 2018; Slominski et al., 2018b).

#### Particulate Matter and Extracellular Matrix

So far, most of the effects of PM have been observed in keratinocytes, but not in dermal fibroblasts *in vitro*. Park in 2018 (Park et al., 2018) reported the overexpression of proinflammatory genes and proteins after exposition to PM_10_. Also, there was a significant increase in cytochrome P450 (CYP1A1, CYP1B1), MMP-1, and MMP-3 along with a decrease in TGF-β, type I collagen. Autophagy, as a cellular stress response, also increased, as demonstrated by TEM (Park et al., 2018). The results show that PM_10_ contributes to the cutaneous inflammatory phenomenon, alterations in the ECM, and altered collagen synthesis (**Table 1**, **Figure 1**).

As mentioned before, PM inflammatory phenomena and oxidative stress induced by PM are the mechanisms involved in skin aging and also the degradation of collagen and the overexpression of MMP.

#### New Receptors in PM and Oxidative Stress/Inflammation

Although it is known that PM produces inflammation and oxidative stress, no keratinocyte receptor has been linked to those responses. However, transient receptor potential vanilloid 1 TRPV1 is known to be expressed in the epidermis, and it can act as a sensitive transducer (Kwon et al., 2018). PM (SRM 1648a, mean particle diameter 5.85 µm) increased expression of the TRPV1 gene. The effects mediated by PM in the expression TRPV1 are due to the activation of p38 MAPK and NF-κB (Kwon et al., 2018). The treatment of cells with a p38 MAPK (AP-1) or NF-κβ inhibitors reduced the increased levels of TRPV 1 induced by PM. The treatment also altered the influx of Ca2+ and cell proliferation, as well as the production of IL-8, TNF-α, and IL-1β (Kwon et al., 2018) (**Table 1**).

In aged skin, *in vitro* and *in vivo* studies have proposed that TRPV1 increases (Lee et al., 2016b). TRPV1 was overexpressed in the sun-protected skin of older subjects compared with younger ones. Besides, the photoaged skin of the elderly showed a higher expression of TRPV1 compared to the skin protected in the same individuals. The increased expression of TRPV1 in aged skin suggests that TRPV1 may be related to senile skin symptoms, such as senile pruritus and inflammation (Lee et al., 2016b) (**Table 1**).

Also, other receptors, such as toll-like receptors (TLRs), have been proposed in the skin for PM-induced ROS production and inflammation. TLRs, the critical receptors for the activation of adaptive and innate immune responses, are expressed in human keratinocytes (Mukherjee et al., 2016). Keratinocytes that appear after the activation of TLRs produce chemokines that, in turn, stimulate the migration of leukocytes to the site of damage (Joo et al., 2012). The binding of PM_2.5_ to TLR5 initiated the intracellular signaling that led the translation of NFκB into the nucleus and the increment of IL-6 (Ryu et al., 2019) (**Table 1**). Besides, PM_2.5_ induced *in vitro* a direct interaction between TLR5 and NOX4 (Ryu et al., 2019). We have also referenced before that NOX increased in photoaging (Tončić et al., 2017).

#### Particulate Matter and Skin Barrier Dysfunction

Skin aging impairs the skin barrier (Kim et al., 2013; Wang and Dreesen, 2018). Additionally, the skin barrier is affected by PM (Pan et al., 2015). When PM_2.5_ is applied to a human three-dimensional skin model, the expression of keratin (KRT) 10, desmocollin 1 (DSC1), and claudin 1 (CLDN1) levels decreases (Pan et al., 2015; Kim et al., 2017). DSC1 is associated with differentiation and keratinization. The “1” isoforms of DSC can provide strong adhesion for damage to keratinized epithelia. *In vivo* studies in BALB/c mice confirm the disruption of the skin barrier by PM (1-μm diameter) (Jin et al., 2018) (**Table 1**).

Recently, in 2019, Nguyen et al. (2019) proposed that skin barrier disruption is due to the apoptotic action PM and the decrease in the keratinocyte proliferation. HaCaT cells and a three-dimensional human skin model exposed to PM (diesel exhaust particles, diameter 10–30 nm) reduced the expression of KRT 16. KRT 16 and 17 are markers of epidermal proliferation and responsible for the mechanical integrity of the keratinocytes. PM-induced apoptosis includes increased p53 and Bax expression (**Table 1**). Additionally, PM stimulation increased the expression of cleaved caspase-3 (Nguyen et al., 2019).

FLG is an essential element of the skin barrier. FLG is vital for keratinocytes to acquire the properties of physical force through the aggregation of the keratin packages of the upper epidermal strata. FLG contributes to epidermal hydration (Lee et al., 2012). PM (SRM1649b, average diameter 10.5 μm) downregulates mRNA and FLG protein in HaCaT cells. PM also induces an increase of COX2 and PGE2 involved in the decrease of FLG (**Table 1**). Pretreatment with inhibitors of COX2 and PGE2 attenuated the decrease of FLG. Also, the transfection with the siRNA, specifically for AhR, reverts the PM mediated downregulation of FLG (Lee et al., 2012).

Similarly, UVA activated NOX activity decreasing FLG expression (Valencia and Kochevar, 2008). Also, a single dose of UVB exposure downregulates FLG, which may explain skin dryness in photoaging (Tončić et al., 2017).

#### Particulate Matter and Human Studies

A recent review has reported the impact of air pollution on humans. Many studies relate to the relationship between air pollution and AD (Krutmann et al., 2017; Burke, 2018). PM represents an important factor that contributes to extrinsic skin aging in humans (Vierkötter et al., 2010; Li et al., 2015; Hüls et al., 2016; Krutmann et al., 2017; Burke, 2018).

However, a relationship between air pollution and skin aging was first shown in the SALIA study (SALIA, *study on the influence of air pollution on lung function, inflammation, and aging*), an epidemiological study of 400 Caucasian women aged 70–80 years, from 2008 to 2009, which indicated that exposure to trafﬁc-related PM contributes to skin aging (Vierkötter et al., 2010). Higher concentrations of PM_10_ were associated with more pigmentation spots on the face and more pronounced nasolabial folds. The distance of residence to the nearest road was also associated with more pigment spots, but this effect did not reach significance (Vierkötter et al., 2010).

Subsequently, studies have been done in Chinese population (Hüls et al., 2016; Peng et al., 2017) partly due to the increasing levels of air pollution, due to the economic development. The association of skin aging and air pollution was analyzed in two areas in Beijing with high and low levels of PM_2.5_, in 400 women with ages that ranged from 40 to 90 years (Peng et al., 2017). The exposition of high levels of PM_2.5_ was significantly associated with skin aging. In the area with high levels of PM_2.5_, the number of senile lentigines spots on cheeks and back of hands was 1.48 and 2.8 times higher, respectively, compared to the population exposed to the lower levels of PM_2.5_ (Peng et al., 2017) (**Table 3**).

Photodamage contributes to the formation of lentigo senile in human skin defined as the result of chronic exposure to a variety of environmental factors including UVR (Krutmann et al., 2014). The Asian population generally avoids sun exposure, and the photodamage is lower than in the Caucasian population (Hüls et al., 2016) (**Table 3**). However, the senile lentigo appears earlier in the Asian population than in the Caucasian population, so it seems that high air pollution could produce an increase in senile lentigo. As shown in *in vitro* and *in vivo* studies, environmental contaminants such as PAH and TCDD and dioxins produce hyperpigmentation by increasing the enzymes that synthesize melanin *via* AhR activation (Luecke et al., 2010).

Also, indoor air pollution has been investigated in humans and epidemiological studies have shown that indoor air pollution causes skin aging (Li et al., 2015). Cooking with solid fuels (such as wood, animal manure, crop residues, and coal) is one of the primary sources of exposure to indoor air pollution in China. The smoke from solid fuels contains thousands of substances that harm human health. The most important are sulfur oxides, carbon monoxide, and polycyclic organic matter such as benzo[a]pyrene (B[a]P). In a population of about 1,200 Chinese women between the ages of 30 and 90, cooking with solid fuels was associated with the occurrence of severe facial wrinkles and fine lines on the backs of the hands, regardless of age and other factors that influence skin aging (Li et al., 2015).

In summary, multiple *in vivo* and *in vitro* investigations appear to demonstrate the role of PM in skin aging, which involves the induction of ROS accompanying with damaged proteins and DNA and derivatives of inflammatory phenomena.

### Persistent Organic Pollutants

Persistent organic pollutants (POPs) are organic compounds that have made a global impact due to their persistence in the environment. POPs come from industrial processes, waste, traffic, and agriculture. Some may be of natural origin, such as those released after volcanic eruptions. POPs can be transported over long distances. They have adverse effects on human health and ecosystems. Bioaccumulation occurs because POP is resistant to natural/environmental degradation (Stockholm Convention, 2011).

Humans are exposed to these chemicals through food and through the air outdoors and indoors. Therefore, POPs can be found virtually anywhere on our planet in measurable concentrations (World Health Organization, 2003). POPs bioaccumulate in organisms, including human body, through stable bonds with lipids and are magnified in the food chain.

Human exposure, even at low POP levels, can, among other things, increase the risk of cancer (Stockholm Convention, 2011). The Convention of Stockholm in 2001 identified 12 POPs or the “dirty dozen.” According to the source, POPs can be grouped into three categories: i) pesticides that, although banned, are still used in some countries, ii) POPs whose origins are from the industry, like polychlorinated biphenyls (PCB), and iii) unintended industrial by-products. The latter include especially dibenzo-p-dioxins (PCDD), polychlorinated dibenzofurans (PCDF), polychlorinated dibenzofurans (PCDFs), and polycyclic aromatic hydrocarbons (PAHs). The chemical name of dioxin is 2,3,7,8-tetrachlorodibenzo (TCDD). However, the name “dioxins” is often used for the family of PCDDs and PCDFs that are related by their structure and chemical composition. They are also called “dioxins” to certain dioxin-like PCBs that have similar toxicity dioxin (Stockholm Convention, 2011).

Exposure to extremely high concentrations of dioxin produces, among others, skin lesions in humans as chloracne. The most extensive studies of the effects of POP in humans are due to the incidents that took place in Japan in 1968, Italy in 1976, Germany in 2010, and more recently, in 2014, when Victor Yushchenko, the former President of Ukraine, was poisoned with TCDD. Although it has been difficult to prove the involvement of POP in aging, the damages produced in the skin and the mechanisms involved seem the same as those that occur in intrinsic and extrinsic aging.

From the POP, PAH is highly lipophilic and penetrates easily in the skin. When PAH is metabolized through the enzymes of phase I, it can be covalently bound to the DNA that forms the PAH-DNA adducts (Froehner et al., 2018). Also, DNA can be damaged by the ROS generated during the metabolic activation of PAH (Tsuji et al., 2011) thus owing high toxicity, mutagenic, and/or carcinogenic effects (Idowu et al., 2019). The United States Environmental Protection Administration (EPA) has included seven PAH in group B2 (probable human carcinogens): benzo anthracene, chrysene, benzo(a)pyrene, benzo(b)fluoranthene, benzo(k)fluoranthene, dibenzo(a,h)anthracene, and indene(1,2,3-cd)pyrene (Working Group on Polycyclic Aromatic Hydrocarbons, 2001).

#### Persistent Organic Pollutants (PAH and TCDD) and Oxidative Stress/Inflammation

The most frequent routes of exposure to PAH are inhalation and skin absorption. Other means of exposure to PAH include food and drinking water contaminated by environmental sources. Within the PAH, B[a]P has been the most studied due to its carcinogenicity in humans (Stockholm Convention, 2011). The toxic equivalency factor (TEF) for PAH is related to one of the most significant carcinogens, B[a]P (Idowu. et al., 2019). PAHs are also photosensitive and absorb sunlight in the UVA range (Working Group on Polycyclic Aromatic Hydrocarbons.Ambient air pollution by polycyclic aromatic hydrocarbons (PAH), 2001; Costa et al., 2010) generating ROS.

Studies *in vitro* have shown the ability of B[a]P’s to produce skin inflammation and oxidative stress (Tsuji et al., 2011; Fuyuno et al., 2018) and how its ligand causes damage to AhR (Tsuji et al., 2011). When the line of human epidermal keratinocytes (NHEK) was exposed to B[a]P at increasing nuclear concentrations of AhR was observed and as a result of an increment of CYP1A and ROS production in a dose-dependent manner (Tsuji et al., 2011). Knockdown AhR expression produces an inhibition of ROS and an IL-8 increase induced by B[a]P; thus, the inflammatory response and the increase of ROS due to B[a]P’s are dependent on AhR signaling (Tsuji et al., 2011; Fuyuno et al., 2018) (**Table 2**) (**Figure 1**).

**Table 2:** Effects of polycyclic aromatic hydrocarbons (PAH), dioxin (TCDD) and Ozone (O_3_) in the skin.

Skin effects	Pollutants	Type of study	Skin damage	Mechanism skin damage	References
**Oxidative stress/inflammation**	PAH B[a]P	*In vitro*	↑ROS ↑IL-8	↑AhR ↑CYP1A1	(Tsuji et al., 2011; Fuyuno et al., 2018)
	TCDD	*In vivo* HRS/*hr/hr* mice	↑Mast cells ↑Macrophages ↑Immune cells ↑ IL1β, IL6, IL22 ↑TNF-α ↑CCN1	↑AhR ↑Expression of proinflammatory genes	(Rudyak et al., 2018)
	O_3_	*In vitro*	↑ROS	DNA breakage mitochondrial damage ↓ ATP ↓Sirtuin	(McCarthy et al., 2013)
		*In vitro*	↑ROS ↑IL-8 ↓keratinocytes proliferation	↑4-HNE protein adduct ↑Nrf2 nuclear translocation ↑NF-kB	(Valacchi et al., 2015)
		*In vivo* SKH-1 mice	↑ROS	↑Lipid peroxidation ↑4-HNE	(Valacchi et al., 2002)

4-HNE, 4-hydroxy-2-nonenal; AhR, aryl hydrocarbon receptor; B[a]P, benzo (a) pyrene; CCN1, cysteine-rich protein 61; CYP1A1, cytochrome P450 (CYP1A1); IL, interleukin; NF-kB, nuclear factor kappa B; Nrf2, nuclear factor erythroid 2-like 2; ROS, reactive oxygen species; TCDD, dioxin/2,3,7,8-tetrachlorodibenzo; TNF-*α*, tumor necrosis factor.

TCDD is responsible for a variety of toxic effects *via* AhR signaling pathways (Patrizi and Siciliani de Cumis, 2018). It induces the translocation of AhR to the nucleus and upstream to the promoter of the CYP1A1 gene as well as other Ah-responsive genes that regulate the expression of proinflammatory genes and genes related to the dioxin metabolism.

It was reported that dioxin induces skin inflammation *in vivo*. Recently, the *in vivo* study published by Rudyak et al. (2018), in HRS/hr/hairless mice reported that TCDD treatment alone leads to increased dermal infiltration (two- to threefold) by mast cells, macrophages, and other immune system cells. TCDD also induced an increase in the expression of pro-inflammatory cytokines and inflammation markers such as IL1β, IL6, IL22, TNF-α, and cysteine-rich protein 61 (CCN1) (Rudyak et al., 2018) (**Table 2**).

In human aged skin, the interaction of CCN1 with αVβ3 integrin has been reported (Quan et al., 2011; Quan et al., 2012). The interactions stimulate MMP-1 collagen fibril fragmentation (Qin et al., 2013). TCDD also interrupts normal healing (Moirangthem et al., 2013) in a murine cutaneous wound healing model (Moirangthem et al., 2013). After exposure to TCDD a decrease in tensile strength was observed in wounds, indicating an aberrant wound healing. TCDD increased the population of macrophages within the tissue in the later stages of healing (Moirangthem et al., 2013). These findings support the idea that exposure to environmental contaminants, including TCDD, is proinflammatory and interrupts normal healing (**Table 2**). Aging also produces a progressive decrease in skin function, leading to the accumulation of age-related pathologies, such as impaired wound healing (Maru et al., 2014).

Dioxin also increases the pigmentation in melanoma cell line FM55 (Luecke et al., 2010) by increasing the expression of the enzymes responsible for its synthesis. The melanin production in the melanocytes is regulated through the expression and activity of the induction of the tyrosinase gene (TYR), and the tyrosinase 1 (TYRP-1) and 2 (TYRP-2) related proteins gene expression (Slominski et al., 2004; Slominski et al., 2012a; Slominski et al., 2018a). The activation of AhR on melanogenesis after exposure to TCDD was previously reported (Luecke et al., 2010).

The adverse effects of POP in the viability of keratinocytes, the inflammatory responses, and the increase of ROS suggest that POP could influence the skin aging and oxidative stress produced in the aged skin.

#### Persistent Organic Pollutants (TCDD) and Human Studies

In humans, “chloracne” is the most surprising toxic phenomenon due to exposure to TCDD. This pathology is characterized by hyperkeratinization and metaplasia of the sebaceous glands. It has been described that cutaneous lesions induced by TCDD are recognized as hamartomas and are found in parallel with atrophy of the sebaceous glands (Mitoma et al., 2015a)

The contamination of “Yusho” refers to a massive poisoning in 1968 in Japan caused by the ingestion of rice bran oil contaminated with polychlorinated dibenzo-p-dioxins, polychlorinated biphenyls, and polychlorinated dibenzofurans. At the time of the incident, patients suffered from different symptoms, including skin alterations (The Yusho Study Group has performed medical examinations every year for more than 45 years.) (Mitoma et al., 2015a) (**Table 3**).

**Table 3:** Effects of the particulate matter (PM), persistent organic pollutants (POP), persistent polychlorinated biphenyls (PBC) and ozone (O_3_) in human skin.

Skin damage/sign	Pollutants	Type of study	Skin damage/mechanism	References
**Oxidative stress/inflammation**	POP Dioxin (A)	Yusho patients (1131 patients) *vs*. general population 2008	↑Prevalence and severity of the skin scars and comedones. Skin damage correlated POP blood levels *vs* general population	(Mitoma et al., 2015b)
	PBC	Germany workers contaminated by PBC	↑IL-1β correlation with PCB levels	(Leijs et al., 2018)
	O^3^ **0.8 ppm**	19 Caucasian volunteers	↑ROS ↑LOOH Depletion of E vitamin	(He et al., 2006)
		15 Caucasian volunteers (forearm)	↑IsoP ↑COX-2. ↑4-HNE ↑NF κB	(Valacchi et al., 2017)
**Skin pigmentation**	PM_10_	400 Caucasian women SALIA cohort	↑Spots on the face association traffic-related airborne particles	(Vierkötter et al., 2010)
	PM_2.5_	G1 210 *vs* G2 190 Asiatic women G1 low levels PM_2.5_ G2 high levels PM_2.5_	↑Lentigines son cheeks and back in G2 *vs* G1	(Peng et al., 2017)
	POP Dioxin (A)	Yusho patients (1313 patients)	↑Skin pigmentation more prevalent in Yusho patients *vs* general populations	(Mitoma et al., 2015b)
**Wrinkles**	PM_10_	400 Caucasian women SALIA cohort	↑Nasolabial folds association traffic-related airborne particles	(Vierkötter et al., 2010)
	O_3_ (B)	BASE I cohort (SALIA cohort): 806 BASE-II cohort Berlin: 1207	↑Coarse wrinkles on the forehead	(Fuks et al., 2019)

4-HNE, 4-hydroxy-2-nonenal; COX, cyclooxygenase; IsoP, 8-iso-prostaglandin-F(2α); NF κB, nuclear factor kappa B; SALIA, study on the influence of air pollution on lung function, inflammation, and aging. (A). Polychlorinated dibenzo-p-dioxins, polychlorinated biphenyls, and polychlorinated dibenzofurans. (B). 17 days of exceedance per year of O^3^ lower than allowed by the EU (25 days/3 years).

In two studies (Mitoma et al., 2015b; Mitoma et al., 2018) it was observed that the mean POP blood concentrations in Yusho patients were higher than those in the controls. The prevalence and severity of the skin scars and comedones were correlated with age (Matsumoto et al., 2013) and POP blood levels (Mitoma et al., 2015b; Mitoma et al., 2018) (**Table 3**). Since the time of contamination, numerous therapeutic strategies have been tried, some of them involving antioxidant phytochemicals and herbal extracts. The Yusho group revealed that several phytochemicals regulate AhR signaling (Mitoma et al., 2018).

After the contamination in Germany in 2010 by PCB, high serum levels of the contaminant were found in workers at a transformer recycling company in Germany. The average life of PCBs and their bioaccumulation properties allowed them to show up even in 2013 blood levels in contaminated workers (Leijs et al., 2018). Between 2010 and 2015, in 25 workers, PCB blood levels and skin samples were studied. In this subgroup, a significant correlation between the upregulation of IL-1β in skin samples and PCB levels was found (Leijs et al., 2018) (**Table 3**). These findings confirm that exposure to PCBs is related to inflammation and that the increase in skin levels of IL-1β might be a risk for developing skin aging.

### Gaseous Pollutant. Ozone (O_3_)

Ozone (O_3_) is a fundamental component of the stratosphere, but it is also found in the troposphere. In the stratosphere, it has a vital role in protecting against the ultraviolet radiation (UVR) of the sun. In the troposphere, it is considered to be a secondary contaminant because it is formed through primary chemical precursor reactions. The majority of tropospheric ozone formation is produced from primary contaminants such as VOCs, nitrogen oxides (NOx), and carbon monoxide (CO), which react in the atmosphere in the presence of sunlight. All of these processes also induce the formation of other products, such as nitric acid and sulfuric acid, and to other compounds, such as formaldehyde (Nuvolone et al., 2018).

The anthropogenic sources of the ozone precursors include industrial emissions, motor vehicle leaks, and chemical solvents. The EPA has established national environmental air quality standards for O_3_ in 0.07 parts per million (ppm) by volume, but O_3_ in urban environments can range from 0.2 to 1.2 ppm (Nuvolone et al., 2018).

#### Ozone and Oxidative Stress

O_3_ is probably the most potent oxidant to which human skin is regularly exposed (Valacchi et al., 2002; Valacchi et al., 2015; Fuks et al., 2019). It produces adverse reactions in the upper and deeper layers of the skin. *In vivo* and *in vitro* studies have shown that short-term exposure to O_3_ produces ROS, biomolecule oxidation, depletion of cellular antioxidant defenses, cell stress, and cytotoxicity (Valacchi et al., 2002; Valacchi et al., 2015; Krutmann et al., 2017). Also, O_3_ induces damage to the extracellular matrix increasing MMP-9 accompanied by a decrease in collagen types I and III (Fuks et al., 2019). Therefore, long-term exposure to tropospheric O_3_ can contribute to skin aging (**Table 2**) (**Figure 1**).

*In vitro* studies evidence that O_3_ causes DNA breakage and depletes the levels of ATP and sirtuin 3, compromising mitochondrial function, and downregulating cell proliferation (McCarthy et al., 2013; Valacchi et al., 2015). Also, the exposition to O_3_ (0.1 ppm) induced not only an increase of the nuclear factor erythroid 2-like (Nrf2) levels and also a greater nuclear translocation as an internal cellular response to the oxidative stress induced by O_3_ (**Table 2**). On the other hand, O_3_ produces lipid peroxidation and damage to the barrier function of the epidermis. *In vivo* studies show that O_3_ exposed SKH-1 mice also reported an increase of 4-hydroxynonenal (4-HNE) (Valacchi et al., 2002).

#### Ozone and Human Studies

He et al. (2006), using a specially designed exposure chamber, provided the first evidence of the human effects of O_3_. Indeed, O_3_ (0.08 ppm, at a level observed in polluted cities) significantly reduced vitamin E and, in the superficial SC, increased lipid hydroperoxides by 2.3-folds. Valacchi in 2017 (Valachhi et al., 2017) exposed the forearm skin of 15 volunteers to 0.8 ppm and showed a significant increase of 4-HNE and 8-iso-prostaglandin-F(2α) (IsoP) compared to non-exposed skin. Meanwhile, O_3_ also caused the activation NF-kB and its translocation to the nucleus and up-regulated COX-2 (Valacchi et al., 2017). This ability of O_3_ to induce the activation of NF-κB in keratinocytes has been previously demonstrated *in vitro* and *in vivo* (Valacchi et al., 2004; Valacchi et al., 2016) (**Table 3**).

Very recently, Krutmann group (Fuks et al., 2019) addressed the effects of O_3_ in the skin in two densely urbanized areas of Germany: the area of the SALIA cohort in Ruhr area, and the BASE-II cohort in Berlin. The study included 806 women from the SALIA cohort and 1,207 men and women from the BASE-II cohort. The participants experienced an average of 17 days of exceedance per year of O_3_, a value slightly lower than the values allowed by the European Union (25 days of exceedances, averaged per 3 years). They found an association between days of O_3_ exposure and coarse wrinkles on the forehead, independent of other environmental risk factors (Fuks et al., 2019) (**Table 3**).

In summary, it has been demonstrated *in vitro* and *in vivo* that O_3_ rapidly oxidizes lipids and proteins in the skin, inducing the production of radical species and triggering oxidative stress. The response to oxidative stress caused by O3 is not restricted to the stratum corneum, but also deeper areas of the skin. O_3_ can activate AhR and transcription factors such as NFkB and inflammatory cytokines. O_3_ also induces degradation of the extracellular matrix mediated by the actions of MMP and by the reduction of collagen synthesis and degradations.

## Photo-Pollutants

The importance of solar radiation and its impact on human health has been discussed previously. Overexposure to UVR is the main environmental risk factor in photoaging and photocarcinogenesis. Likewise, environmental pollution is also essential to skin aging and skin diseases. In this regard, it is very relevant to know the impact of UVR, the characterization of the UV spectrum, and the effect of atmospheric pollutants on UV radiation for all environmental health research programs that are being carried out in several countries (Bais et al., 2018) (**Table 4**). For these programs to be efficient, it is necessary to know and appreciate in depth the mechanisms that are triggered in the skin after these environmental aggressors, related to HPA (Skobowiat et al., 2011; Skobowiat et al., 2013; Slominski, 2015; Skobowiat et al., 2017)

**Table 4:** Synergistic effects of UVA and UVB radiation and benzo(a)pyrene (B[a]P) and particulate matter (PM) in the skin.

Skin effects	Pollutants	Type of study	Skin damage	Mechanism skin damage	References
**Oxidative stress/inflammation**	B[a]P +UVA	*In vitro*	↑DNA damage	↑Phosphorylation H2AX	(Toyooka and Ibuki, 2006)
	B[a]P +UVA	*In vitro*	↑ROS	↑Lipid peroxidation	(Xia et al., 2015)
	B[a]P +UVA1	*In vitro*	↑ROS	↑Mitochondrial superoxide ↑Mitochondrial membrane depolarization	(Soeur et al., 2017)
**Pigmentation**	PM+UVB	799 women SALIA cohort	Lentigines		(Hüls et al., 2018)

H2AX, histone H2AX; ROS, reactive oxygen species; SALIA, study on the influence of air pollution on lung function, inflammation, and aging.

Oxidative stress and inflammation are the harmful cutaneous effects of environmental pollution, and more specifically of air pollution. These effects could be amplified by the damaging synergy of the sun, especially UVA rays and pollution. Therefore, protection strategies should combine cutaneous protection by sunscreen with high absorption of UVA and antioxidants providing a greater resistance of skin to oxidative stress and inflammation.

As mentioned before, solar UVR is a carcinogen and the leading cause for skin cancer. PAH is photosensitive and absorbs sunlight in the UVA range, so PHA is excited to higher energy states and, therefore, generates ROS (1107, Marrot, 2018). Even in PAH concentrations below toxic or tumorigenic levels, the combined impact of these two factors seems to contribute to increased tumorigenicity in animals (Wang et al., 2005; Burke and Wei, 2009). After exposure to UVA, the phototoxic power of PAH is demonstrated as PAH decreases the 80% cell viability (Wang et al., 2005; Toyooka and Ibuki, 2006) inducing phosphorylation of histone H2AX, a marker of DNA double-strand breaks.

In a recent review, Burke reported that B[a]P, even at low levels when combined with UVA, contributes to increased oxidative damage in the skin, with an increase in extrinsic aging and tumorigenicity (Burke and Wei, 2009). *In vitro* (Saladi et al., 2003) and *in vivo* experiments have shown that these two carcinogens further elevate ROS levels and oxidative damage to DNA.

B[a]P and its intermediates can act as UVA photosensitizers producing ROS that forms 8-hydroxy-2’-deoxyguanosine (8-OHdG) (Saladi et al., 2003). Besides this, UVA accelerates the cellular metabolism of B[a]P, which leads to a higher increase of B[a]P derivatives. These B[a]P derivatives increase DNA oxidative damage (Saladi et al., 2003) altering signaling pathways and the expression of genes involved on cell survival, extrinsic aging, and photocarcinogenesis. Also, B[a]P metabolites produced lipid peroxidation when photoirradiation was done in the presence of lipids. The formation of lipid hydroperoxides was inhibited by superoxide dismutase. Meanwhile, electron spin resonance indicated that singlet oxygen and a superoxide radical anion were generated from UVA photoirradiation of B[a]P products in a light-dose responding manner (Xia et al., 2015) (**Table 4**).

In addition, studies performed in NHEK have confirmed that photo-pollution is more dangerous than by pollution alone (Soeur et al., 2017). NHEK exposed to daily UVA1 (350–400 nm) combined with PAH has shown significant synergistic phototoxicity damage. Of the PHA, B[a]P, and indeno[1,2,3-cd]pyrene (IcdP) showed the highest phototoxicity at low concentrations (nanomoles per liter). Exposure to PHA and UVA1 decreased the clonogenic potential of the cells and increased ROS. Therefore, the normal epidermal cell cycle can be affected in the chronically exposed skin of photo-pollution, which may worsen the photodamage already induced by UVA1 (Soeur et al., 2017). Also, IcdP and UVA1 affected mitochondria by triggering superoxide production and membrane depolarization. Mitochondrial deterioration induced by contamination and UVR should be considered as a relevant mechanism involved in synergistic skin effects (Soeur et al., 2017) (**Table 4**).

Also, traffic-related air pollutants (TRAPs) have an impact on the deleterious effects of UVR in the skin. TRAP is a mixture of gases and particles that interact with each other and are a source of secondary pollutants. TRAP is involved in the formation of facial lentigines (Vierkötter et al., 2010; Hüls et al., 2016). In the study conducted in the cohort of 799 elderly German women (SALIA), the interaction of UVR and TRAP in the formation of facial lentigines was investigated. This cohort has been used in previous studies on the association between aging skin and air pollution (Vierkötter et al., 2010; Vierkötter et al., 2015; Hüls et al., 2016). The average 5-year exposure to UVR has been estimated using NASA satellite data and data from the German Climate Center. There was a positive association between PM and UVB-whole day and the formation of lentigines (Hüls et al., 2018) (**Table 4**). The study confirms the synergistic effect in the formation of the lentigines of the pollution (TRAP) and UVR.

Finally, and recently, the Hoseinzadeh group in 2018 (Hoseinzadeh et al., 2018) has assessed the synergic effect of air pollution and ultraviolet B radiation in the impact on vitamin D (VD) deficiency (Hoseinzadeh et al., 2018). VD is an essential nutrient to prevent several chronic diseases, and the deficiency of the derivatives of the VD causes many diseases (Bikle, 2008; Bikle, 2012). The protective effect of the active form of vitamin D3 (1,25(OH)2D3) on the damage induced by ultraviolet B radiation is well known (Chaiprasongsuk et al., 2019).

Air pollution seems to be a significant factor that causes the deficiency of the VD derivatives (Hoseinzadeh et al., 2018). Air pollution affects VD levels by reducing sun exposure, especially UVB radiation. Sun exposure accounts for more than 90% of VD production in humans (Holick, 2017). The Hoseinzadeh studies have shown that tropospheric O_3_ and PM are independent risks for decreasing the levels of VD and cause deficiency of this vitamin. The highest deficiencies in vitamin D have been found in the Middle East, Asia, and Northern Europe, respectively. However, it is still necessary to obtain exhaustive conclusions about the impact of air pollution on VD deficiency. Equally, although the effects of photoprotective properties of the VD derivatives (Dixon et al., 2007; Song et al., 2013; Cashman, 2015) have been demonstrated, it is necessary to study in more detail the effect of these derivatives as a therapy against environmental pollution (Hoseinzadeh et al., 2018).

### Concluding Remarks

The research reference in this review shows that air pollution, especially PM, PHA, and ozone, causes premature skin aging, as evidenced by the appearance of wrinkles, senile lentigo, and dry, itchy skin after exposure to air pollutants (**Table 5**). It has been shown in the studies that (Birch-Machin and Bowman, 2016) ROS plays a central role in the responses as air pollutions damage DNA, mitochondria, protein, and lipid membranes (Krutmann et al., 2017). PHA, PM, and O_3_, after union to AhR and its translocation to the nucleus, generate toxic and reactive intermediates, which unbalance redox homeostasis, increasing ROS (Salthammer et al., 2018); in addition to the canonical AhR pathway, the activation of AhR often affects other signal transduction pathways, including NF-κB signals (World Health Organization, 2018). NF-κB signals increase the expression of proinflammatory genes, MMP inductions, and disbalance in collagen formation (World Health Organization, 2012), and pollutants also disrupt the skin barrier and alter the immunity of the skin. Oxidative stress and DNA damage induce keratinocyte apoptosis, decrease epidermal renewal, and delete wound healing. All these phenomena affect not only the keratinocytes but also the dermal fibroblasts, responsible for the renewal of the ECM. Subsequently, the inflammatory processes continue to increase ROS and produce a more significant deterioration of the ECM. PM, PHA, and O_3_ also have a synergistic effect with solar radiation, so photodamage increases.

**Table 5:** Common mechanisms and effects observed in skin aging (↑) and in damage skin after air pollution exposition (▲).

Mechanisms		Biological effects		Effects on the skin	
	Aging/pollutants		Aging/pollutants		Aging/pollutants
Activation of AhR	↑/▲	Increase of melanogenesis	↑/▲	Hyperpigmentation	↑/▲
Generation of ROS	↑/▲	DNA damage. Lipid peroxidation.	↑/▲	Wrinkles	↑/▲
Induction of inflammatory cascade	↑/▲	Decrease collagen	↑/▲	Delayed healing skin	↑/▲
Disruption of skin barrier	↑/▲	Increase MMPs	↑/▲	Dry skin	↑/▲
				Exacerbation of skin diseases	↑/▲

The mechanisms that have been revealed *in vivo* and *in vitro* and the lesions found in humans are of great importance to prevent the premature aging caused by air pollution since the skin elements that are affected by the pollutants could be therapeutic targets in the prevention of aging and/or the pathologies associated with skin aging.

## Contribution to the Field Statement

Aging increases the likelihood of skin lesions. Aging is due to extrinsic and intrinsic factors. Until now, great emphasis has been placed on the harmful effects of solar radiation on the skin. However, it is not only solar radiation that causes damage. The quality of the air breathed is a global concern, and according to the estimates of the World Health Organization, 9 out of 10 people breathe contaminated air. In this review, we describe, based on *in vitro* studies, in animals and in humans, the mechanisms that cause damage due to air pollutants in the skin and how they influence skin aging. Oxidative stress, inflammation, and damage of the cutaneous barrier and in the extracellular matrix are effects of pollution and are observed in skin aging. The cellular and nuclear factors affected in the epidermal and dermal cells by contaminated air are also altered in aging. Therefore, aging caused by the environment is currently a fundamental element to take into account in health. Besides, the synergistic effect of sunlight and environmental pollution on the skin cannot be ignored. The search for preventive treatments against the synergy of sunlight and environmental pollution is of vital importance for healthy skin.

## Author Contributions

CP, YG, SG, and AJ substantially contributed to the conception and design of the study. SM and AP-D organized the data base acquisition for the referenced research *in vivo* and *in vitro* studies. All the authors have contributed to the analysis of data for the work, revising it critically for important intellectual content. All the authors provided approval for publication of the content, contributed to manuscript revision, and read and approved the submitted version. The corresponding author SG has finally approved all parts of the final version of the manuscript and has the same level of responsibility as AJ.

## Funding

This research was funded by Spanish grants from Instituto de Salud Carlos III MINECO and Feder Funds (FIS: PI15/00974, PI18/00708, and PI18/00858).

## Conflict of Interest Statement

The authors declare that the research was conducted in the absence of any commercial or financial relationships that could be construed as a potential conflict of interest.
